# Developing interprofessional health competencies in a virtual world

**DOI:** 10.3402/meo.v17i0.11213

**Published:** 2012-11-16

**Authors:** Sharla King, David Chodos, Eleni Stroulia, Mike Carbonaro, Mark MacKenzie, Andrew Reid, Lisa Torres, Elaine Greidanus

**Affiliations:** 1 Department of Educational Psychology, University of Alberta, Edmonton, Canada; 2 Department of Computing Science, University of Alberta, Edmonton, Canada; 3 Department of Emergency Medicine, University of Alberta, Edmonton, Canada; 4 Alberta Health Services, Edmonton, Canada; 5 Northern Alberta Institute of Technology, Edmonton, Canada; 6 Health Sciences Education Research Commons, University of Alberta, Edmonton, Canada

**Keywords:** virtual worlds, simulation, interprofessional education, development framework

## Abstract

**Background:**

Virtual worlds provide a promising means of delivering simulations for developing interprofessional health skills. However, developing and implementing a virtual world simulation is a challenging process, in part because of the novelty of virtual worlds as a simulation platform and also because of the degree of collaboration required among technical and subject experts. Thus, it can be difficult to ensure that the simulation is both technically satisfactory and educationally appropriate.

**Methods:**

To address this challenge, we propose the use of de Freitas and Oliver's *four-dimensional framework* as a means of guiding the development process. We give an overview of the framework and describe how its principles can be applied to the development of virtual world simulations.

**Results:**

We present two virtual world simulation pilot projects that adopted this approach, and describe our development experience in these projects. We directly connect this experience to the four-dimensional framework, thus validating the framework's applicability to the projects and to the context of virtual world simulations in general.

**Conclusions:**

We present a series of recommendations for developing virtual world simulations for interprofessional health education. These recommendations are based on the four-dimensional framework and are also informed by our experience with the pilot projects.

Providing safe and quality care requires health professionals to possess skills for teamwork and collaborative practice, specifically interprofessional (IP) collaboration and communication (1–3). Therefore, health educators have a responsibility to ensure students graduate with the competencies to work effectively in a team. The development of collaborative team skills requires an intentional focus on providing IP learning experiences that immerse students in safe environments where learning – and mistakes – can occur. One approach is to create an IP simulation environment that allows students to develop relevant clinical skills (4). Simulation involving computer-based training, mannequins, task trainers and standardised or simulated patients is an established educational learning and assessment method in health science education (5, 6). More importantly, in the area of IP education, simulation has been identified as a *model of best practice* for teaching and learning (7).

Despite the benefits to IP simulation as a learning tool, challenges exist. For example, IP team-based simulation scenarios are often constrained by temporal and geographic challenges when students are required to meet face-to-face for the interaction. Furthermore, resources and logistics required to deliver a single, complex scenario such as disaster training, for example, may be substantial and prohibitive in terms of cost (8).

Overcoming these challenges has led IP education researchers to expand the use of health science simulations to investigate the possibility of using virtual worlds (VWs) (9, 10). VWs are computer-based three-dimensional simulation environments that usually take the form of online communities. Individuals appear in these environments in the form of avatars (i.e., personified computer characters) and interact with each other and their three-dimensional graphical environment. Such environments are reminiscent of computer video games, and there are strong arguments for the potential of VWs to support and enhance learning (11).

In the context of IP education, the use of VWs to enhance clinical and teamwork skills for health science students and practitioners offers many possibilities. VWs may be used to provide content in a relatively static manner, facilitate discussions and interactions, simulate patient care in environments such as emergency rooms, operating rooms and hospital wards (12) or to assist with disaster planning (13, 12). Using a VW as a simulation platform provides three key benefits. First, VWs enable geographically dispersed people to come together for real-time interactive training situations that would be too expensive to implement in a real-world setting. Second, one can develop VW environments that students would not be able to access easily in the real world (e.g., international locations). Third, one can reuse the same VW environments repeatedly to train students in high-risk, low-frequency situations.

Designing, developing and evaluating an appropriate pedagogical approach is critical for the successful implementation of VW educational scenarios (14, 15). The development of a VW for instructional purposes requires collaboration between VW designers, content experts and education specialists (16), as shown in Fig. 1. In order to address these complexities, de Freitas and Oliver (17) proposed a four-dimensional framework for evaluating simulation-based education. According to de Freitas and Oliver, the framework allows ‘educational designers to consider a more user-based and specialized set of educationally specific factors’ (p. 253) that can be employed to guide the design of VW-based learning environments.

**Fig. 1:**
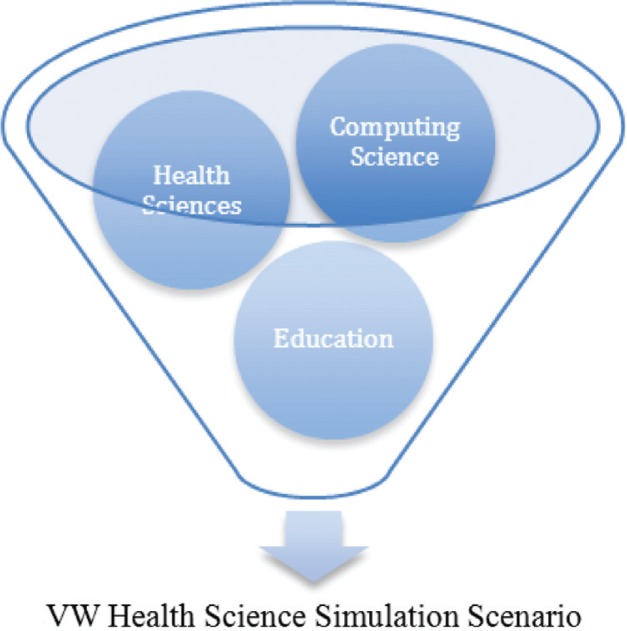
Collaborative scenario development process.

The purpose of this paper is fourfold. First, we outline de Freitas and Oliver's *four-dimensional framework* (17) and use this framework to guide our pedagogical approach in developing VW scenarios. Second, we describe the development of the IP educational health team simulation scenario that was implemented in a VW and how this simulation was mapped onto the dimensional framework. Third, we describe the pilot implementation and results from two of the simulation scenarios: (a) a rescue simulation, involving students from a paramedic program and emergency room physicians; and (b) a revised version of the scenario that expanded the IP contribution to include emergency nursing students. Finally, we conclude with a discussion and lessons learned from our analysis on the use of the de Freitas and Oliver (17) framework for VW simulation education and recommendations for implementation in the IP health sciences education.

## Framework for evaluating simulation-based education

The framework proposed by de Freitas and Oliver (17) was developed through an extensive review and by applying it in a number of test situations. Three questions were identified as critical first steps in the framework creation: (a) what is the appropriate game or simulation for a specific learning context? (b) what is the appropriate pedagogical approaches to use to support learning outcomes and activities? and (c) what is the educational validity when using games or simulations? Educators constructing their own VW simulations can use these questions to guide their design and development in conjunction with the following four dimensions:

1. The first dimension is concerned with the *context* of where the learning occurs and whether or not it is formal or informal. The view of context, in this framework, is multi-levelled and is concerned with the learning environment, the tools that support learning, and the relationship between context and practice (i.e., the VW and the real world).
2. The second dimension, *learner specification*, starts with identifying who the learner is (background, history), learning style and whether there is more than one learner (i.e., a team or group). If the learner specification does involve a team/group, then identifying how learners collaborate to accomplish their goals is essential. The learner-centred focus highlights the importance of the interaction between the learners and their environment and the need to ensure that learning activities match required outcomes. Learner feedback and time for reflection are critical components within this dimension.
3. *Internal representation of world*, the third dimension, involves the level of fidelity and immersion required for the experience to achieve the learning outcomes and the expected level of interactivity.
4. The fourth dimension, *pedagogic considerations*, includes models of learning, such as associative, cognitive or social/situative. The learning theory selected will influence the type of learning outcomes. The selected models may focus on task-centred approaches or socially constructed approaches.

To assist developers, de Freitas and Oliver provide a checklist that designers or evaluators can apply to educational games and simulations (Table 1). Note that the index numbers are used for cross-referencing information in Table 2.

**Table 1:** Framework checklist

Context	Learner specification	Pedagogic considerations	Mode of representation
1. What is the context for learning? (e.g., school, university, home)	1. Who is the learner?	1a. Which pedagogic models and approaches are being used? 1b. Which pedagogic models and approaches might be the most effective? 1c. What are the curricula objectives?	1. Which software tools or content would best support the learning activities?
2. Does the context affect learning? (e.g., level of resources, accessibility, technical support)	2a. What is their background and learning history? 2b. What are the learning style/preferences? 2c. Who is the learner group?	2. What are the learning outcomes?	2. What level of fidelity needs to be used to support learning activities and outcomes?
3. How can links be made between context and practice?	3. How can the learner be best supported?	3. What are the learning activities?	3. What level of immersion is needed to support learning outcomes?
	4. In what ways are the groups working together (e.g., singly, partially in groups) and what collaborative approaches could support this?	4. How can the learning activities and outcomes be achieved through existing games or simulations?	4. What level of realism is needed to achieve learning objectives?
		5. How can the learning activities and outcomes be achieved through specially developed software? 6. How can briefing/debriefing be used to reinforce learning outcomes?	5. How can links be made between the world or the game/simulation and reflection upon learning?

Source: Ref. (17, p. 256).

**Table 2:** Application of de Freitas and Oliver framework to our VW simulation

Context	Learner specification	Pedagogic considerations	Mode of representation
1. Learning context: post-secondary education in health sciences. Designed for at home or classroom-based	1. Students from paramedic program, medicine, nursing, respiratory therapy	1. Theories used include Brown's situated cognition and Kolb's experiential learning theory	1. Second Life best supports the collaborative learning activities, as indicated by comparative analysis of simulation technologies
2. Effect of context on learning: some technical support required initially	2. Learners have range of backgrounds, learning styles and preferences	2. Learning objectives: communicate effectively for patient safety Demonstrate effective verbal and non-verbal communication for patient safety Communicate effectively in transitions in care to ensure the safety of patients Use effective written and verbal communication and communication technologies to provide safe patient care	2. The focus is on developing interprofessional communication in environments that many novice learners would not experience before entering clinical practice
3. Integration into curriculum will follow development of the VW	3. Learner can be supported by providing opportunities for relevant, realistic simulation of learning objectives	3. Learning activities: transport victim from collision scene to emergency room and hand victim off to trauma team	3. Level of immersion: students must fully participate in rescue experience, and be able to conduct conversations within adopted roles (EMT team member, ER staff)
	4. The scenario requires students to work in interdisciplinary teams to collaboratively complete the scenario	4. Existing simulation: can use real-world simulation with standard patients and simulated equipment	4. Level of realism: students must be able to complete rescue tasks, communicate effectively, and feel immersed in the scenario
		5. Special software: can use virtual world simulation, participants use avatars	5. Links between simulation and reflection: students participate in a post-simulation debriefing session with instructors
		6. Simulation designed to include a debriefing after the experience to allow students to reflect on the experience	

## Scenario development in the VW: applying the framework

The learning objectives for our project were to develop IP communication and collaboration competencies. The objectives were revised from the Canadian Patient Safety Institutes’ Safety Competencies (2) and included the domains that focused on communication and teamwork. The desired outcome was to develop a scenario that included disciplines, paramedic, medicine and nursing, that typically did not work together in a highly complex environment prior to entering clinical practice.

The next step was to develop a relevant, complex scenario involving these disciplines. It was determined that an automobile collision scene, requiring transport of the victim to the hospital, including the hand-off of the victim to the trauma team, would be a realistic and relevant scenario that engaged these three disciplines. This scenario was selected for development in a VW for two reasons. First, the complexity of simulating the learning environment to replicate a car collision and hand-off to trauma team using mannequins or simulated patients would have been extremely challenging (i.e., expensive) and not easily reproducible; in other words, the VW simulation scenario, once built, can be reused many times with a variety of student cohorts. Second, the disciplines (paramedic, medicine and nursing) involved in this scenario are educated at institutions in different geographic locations and do not have the opportunity to work together prior to clinical practice. The scenario focuses on both technical skills and human factors, such as communication and collaboration (18).

**Fig. 2:**
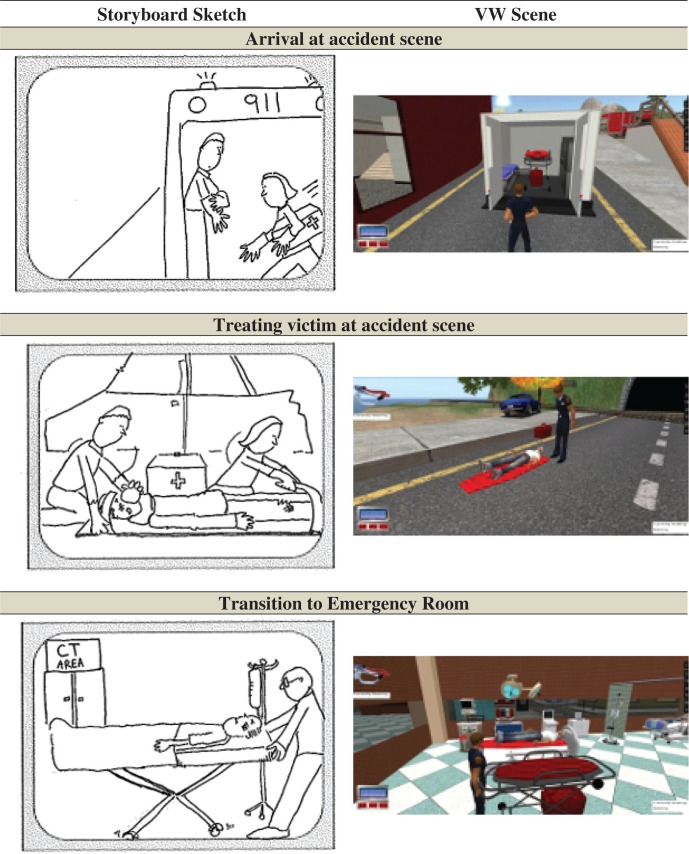
Sample storyboard scene and corresponding VW scene.

After the setting and the disciplines involved in the scenario were determined, a storyboard (Fig. 2, left) was developed with the content experts to identify key features and learning objectives of the scenario. This information was provided to the collaborators in computing science to begin the development of the VW-based scenario. The VW-based scenario was, as much as possible, based on the storyboards, with the scenes represented in the storyboard recreated in the VW (Fig. 2, right). To aid in the development of the VW simulation and to gain a more in depth understanding of the clinical features of the scenario, the computing science developers visited a trauma bay and ambulance with an emergency physician and paramedic. Further details of the scenario development and implementation process, focusing on the software system supporting the scenarios, have been published elsewhere (19, 20).

### Selecting the VW platform

After a review of the characteristics of current virtual environments (21), Second Life (SL) was selected as the platform to create the simulation. SL is an online VW developed by Linden Labs. Participants within SL are called avatars. Avatars have the ability to participate in individual or group activities, transport themselves to different environments within SL or interact with a range of virtual objects. In short, SL creates opportunities to bring people together in environments that would not normally be accessible to them. Built into the software is a three-dimensional modelling tool, based around simple geometric shapes, that allows a resident to build virtual objects. Detailed information regarding the technical aspects of the environment is published elsewhere (22).

### Application for the four-dimensional framework to VW scenario

Table 2 demonstrates the application of the four-dimensional framework checklist for the development and evaluation of the VW as an educational experience in our study. In order to integrate the framework into this study, the content experts collaborated to discuss each of the four dimensions and the specific applications to this simulation scenario.

## Pilot testing the VW simulation scenarios

An exploratory approach was determined as most appropriate for this work. Two separate cohorts were identified for two pilots. Pilot One involved four paramedic students and two emergency medicine physicians; Pilot Two involved 14 students from a range of disciplines: medicine (two), nursing (six), respiratory therapy (two), pharmacy (two), speech language pathology (one) and diagnostic medicine sonography (two). The pilots were structured to evaluate the (a) usability of the VW environment for students with no VW experience; (b) learning effectiveness of VW simulation scenarios; and (c) instructional framework for subsequent integration into mainstream curriculum.

### Orientation to VW environment

In advance of the first pilot session, students were provided with a training area in SL where they were encouraged to explore and play. The scenario assumed that the participants had no previous experience with SL, aside from the skills taught in the training area. The training area helped the students learn how to move one's avatar, control where the avatar was looking (including zooming in on specific objects) and interact with other avatars and interactive in-world objects. Student participation in the training area prior to the pilot was voluntary. For the second pilot, a formal training area was once again provided for the students, and training time was included at the start of each session.

## Pilot One

For the initial pilot of the module, a paramedic instructor at a polytechnical institute recruited four paramedic students to participate. Students worked in pairs, with each pair running through the experience one time. Two emergency medicine physicians served as the trauma team and were situated in the virtual emergency room. The paramedic instructor also participated as an observer during the pilot.

The VW session was designed in a similar manner to a typical non-VW simulation learning experience, which uses physical objects (mannequins) and standardized patients. The session began with a pre-brief within the VW to familiarize participants with features and work out technical issues, continued with the simulation experience and finished with a debriefing session about the experience. The sessions were recorded using Camtasia. Comments and feedback were collected online from all participants following the session. The recorded notes from the debriefing session were transcribed, and a thematic analysis was completed to identify key themes.

Prior to the start of Pilot One, all participants were emailed instructions for signing into SL, creating an avatar and providing consent. Immediately prior to the session, computing science instructors worked with participants to check audio and to troubleshoot configuration problems.

### Results for Pilot One

The first pair of students who participated in the scenario experienced challenges related to the user interface. These technical challenges resulted in limited transmission of information to the emergency physicians, as little clinical information was obtained by the students. The second pair of students overheard the first pair interacting in the environment. Therefore, they were able to learn from the first pair's mistakes and move through the simulation more smoothly, resulting in a more productive hand-off communication with the emergency physicians.

The debriefing occurred in-world with all participants. The structure of the debriefing sessions included feedback on the technical challenges and discussion of the novelty of the learning environment, as well as exploration of team interactions. Two main themes arose from the feedback session: (a) benefits of the environment for IP communication/collaboration; and (b) technical issues with the environment.

### Benefit of environment for IP communication/collaboration

Participants felt that the experience of having a conversation with actual emergency doctors during a patient hand-off was valuable and would be difficult for students to experience in real life. The doctors felt that the tool could be used in distance education and in training practitioners about new clinical guidelines. It was also expressed that this environment was an improvement over existing communication methods, such as video-conferencing.

The physical interaction between doctors and paramedics in the emergency room (ER) was an unplanned, yet quite meaningful, part of the scenario. On one hand, being positioned far away from the victim left participants feeling left out. However, the paramedics were concerned about being in the way of the doctors, and thus did not want to be too close to the victim after bringing him to the ER. The paramedics needed to manoeuvre their avatars while they conducted the hand-off conversation, in order to stay close enough to the victim while staying out of the way of the doctors’ avatars. This interaction was not among the expected educational outcomes but was highlighted by the students as an important part of the simulation experience, since it closely reflected the actual interaction that would normally take place in the real world.

### Technical issues

The user interface was challenging to use at times, especially for the first group, which made it difficult to treat the patient and also decreased the sense of realism. Despite the technical problems that were encountered and the stress of interacting with a doctor without knowing the necessary clinical information, students felt the experience was valuable. In particular, the student–doctor interaction was meaningful in part *because* it was stressful – the students felt that this stress added to the realism of the scenario.

### Pilot One post revisions

The key revisions that occurred after the pilot focused on technical aspects of the environment. Specifically, students had trouble with the interface of the environment, experiencing difficulty with handling the stretcher and assessing the patient's vital signs. Through this experience, ‘holes’ were discovered in the system logic, which allowed unexpected and unrealistic behaviours to occur during the pilot. In addition to addressing the holes and interface issues, further consultations with the content experts were undertaken to enhance the clinical learning opportunities (procedural and communication skills) within the environment. Specifically, three key issues were addressed: (a) providing an adequate range of treatment capabilities and diagnostic information; (b) ensuring that the information available through the SL simulation was consistent with what students would receive through conventional (in person) simulation; and (c) providing a communication interface during the rescue process that was appropriate for the intra- and interdisciplinary communication that would occur.

To meet these requirements, several improvements were made to the SL scenario over a period of several months. One of these was working closely with the instructors to ensure that the content conveyed by the scenario was indeed appropriate, comprehensive and consistent with educational practices. To this end, the information available was expanded to include more specific areas of analysis and additional diagnosis equipment. The scenario was also expanded to include contextual and environmental information, thus giving the students a more complete picture of the simulated situation.

In addition to these content-based improvements, several other, broadly-focused changes were made. To address usability concerns, several improvements were made to the user interface. First, a custom user interface was designed to facilitate students' wearing and using medical equipment. Second, the way that students interact with objects was simplified, so that the student can always left-click to interact with objects. Previously, students needed to left-click to perform some actions, and right-click for others, which had caused a significant amount of confusion.

On a similar note, changes were made to the student set-up and training process, to help students become familiar with SL. First, the researchers created avatars for students to use while participating in the scenario, rather than asking students to create their own. This ensured that the avatars were set up with the necessary clothing, access privileges and user interface components. The researchers also created a separate, dedicated training area, to give students training in basic SL skills (such as moving, communicating and interacting with objects) and experience in using equipment related to the scenario.

Finally, the action recording system, which tracks students’ behaviour while going through scenario, was improved to provide a more detailed, comprehensive record of the students’ actions. Specifically, the recording system tracks each student's movement, text chat conversation and interaction with objects.

## Pilot Two

The second pilot took place several months later, after the revisions from the previous pilot occurred. The second pilot used a different scenario, although the setting (a hospital ER) was quite similar. In this case, the scenario (based on a mannequin-centric scenario from METI (23)) involved a middle-aged woman who arrived at the ER complaining of shortness of breath. Through simple diagnostic tests (e.g., stethoscope, BP cuff) and conversation, the student is expected to be able to make a diagnosis of asthma and can then recommend medication for the patient.

To develop this scenario, the researchers created several new objects in the VW, including a stethoscope that plays a sound clip of the patient's breathing. However, the vast majority of the scenario implementation, including the simulation engine, student action recording tools, and simulated environment, were re-used from Pilot One. Moreover, the patient object for Pilot Two, which is central to both Pilots One and Two, was developed based on the corresponding patient from Pilot One, with appropriate modifications made for the medical context and specific scenario requirements. Thus, although the medical context of the scenario is different from Pilot One, in terms of the technical development of the scenario, Pilot Two represents a modification of Pilot One, rather than an entirely new scenario. The VW scenario was reviewed by two nursing educators to ensure that it was clinically valid and educationally appropriate.

The students in the second pilot were participating in a day-long event that included mannequin-based and simulated patient scenarios. Students were asked to try out the VW either on their own or in small teams (depending upon the number of participants in each session). In the teams, students collaborated to arrive at a consensus regarding the patient's diagnosis and treatment. In doing so, the students shared their own discipline perspective and learned about other disciplines’ roles and perspectives. The students were provided with a brief training session before they participated in the simulation. After the experience, students were asked to complete a satisfaction survey, respond to questions about the experience and to share their perspective on the potential for learning in this environment. The satisfaction survey was developed by the project team and was scored using a Likert scale with values from 1=strongly disagree to 5=strongly agree.

### Results for Pilot Two

Fifteen students from medicine, nursing, respiratory therapy, pharmacy, speech language pathology and diagnostic medical sonography programs participated in the second pilot. All but four were in the first 2 years of their program. Eleven females and four males participated, with an average age of 27.9 years. None of the students had previous experience with this environment.

Table 3 outlines the satisfaction responses from the students. Students were also asked for their thoughts about the learning experience. Students appreciated the ability to visualize the ER setting in a low-pressure situation. The environment made the students think through the situation systematically and demonstrated to the students their strengths and areas for improvement. Students appreciated the benefit that this VW provided in listening to input from other disciplines and highlighted the importance of communication as a team. Students felt that this VW would be a great learning resource as preparation for non-VW scenarios for clinical situations.

**Table 3:** Student satisfaction survey results from pilot two (Means and SDs)

Questions (1=strongly disagree; 2=disagree; 3=neutral; 4=agree; 5=strongly agree)	Mean
This experience has improved my interprofessional teamwork skills	3.4
The level of realism was sufficient for suspension of disbelief	3.5
The level of realism was sufficient to enable learning	3.8
I was able to apply the following knowledge and skills in completing the scenario:	
Knowledge of medical facts	3.8
Knowledge of relevant procedures	3.8
Communication skills	3.4
The experience was interesting and I felt engaged in the experience	4
I would recommend this experience to other learners	4.1

## Recommendations and discussion

Given the complexity of this project and its novel nature, an exploratory approach was undertaken by the researchers. The project required collaboration among the members of the design team (health sciences experts, educators and computer scientists). The project identified a suitable educational framework to guide the VW simulation development and then scenarios were implemented for two groups of health science students.

To establish a solid pedagogical foundation for the VW scenarios, the *four-dimensional framework* (17) was chosen. The framework and its corresponding checklist provided a structural support to guide the project's design and development. The process of mapping the checklist to the actual scenario provided a level of confidence that we were appropriately applying the VW technology.

### Recommendations

The following recommendations are based on our experience of using the framework to guide the development process for the two pilot studies.

1. *Invest sufficient time at the beginning of the development process to address the details of your design*. The checklist provides an excellent starting point for this design specification, especially for educational aspects of the scenario such as the characteristics of the learners, the pedagogic approaches that will be used and the learning outcomes.
2. *Use storyboards as an extension of the checklist to help map out your scenarios*. Storyboards help the design team think through the details of each phase of the scenario and ensure they can communicate clearly, without becoming mired in context-specific terminology or technical jargon.
3. *Use the checklist's ‘mode of representation’ questions to help address issues of fidelity and immersion*. These issues can be difficult to articulate, especially when bridging gaps in expertise. Computing science researchers are familiar with the representational and immersive capabilities of the VW but are unfamiliar with the requirements of specific health sciences disciplines. For health science educators, the situation is reversed. The mode of representation questions can provide a common starting point for discussion and, thus, help bridge this gap.

The two pilot implementations, a rescue simulation and a revised version of the scenario in an ER context, provided a proof of concept for this project. Bringing the education scenario to life in the VW proved challenging for many reasons. Most critical was developing effective communication and collaboration strategies within the multi-disciplinary design team.

To ensure effective communication and collaboration within the design team, we adopted several strategies, listed below.

1. *At the beginning of the development process, establish a common understanding of the system requirements, educational goals and VW capabilities*. This will help ensure that all team members are on the same page and that each person's expectations (particularly in areas with which they have little familiarity) are in keeping with the capabilities and context of the system.
2. *Hold frequent meetings with team members from each discipline*, to ensure that the system is proceeding acceptably from the point of view of all members and that the requirements and concerns of all members are adequately addressed.
3. Once the system has been sufficiently developed, *test the system with a range of users*, to ensure that a broad range of execution paths are tested, a variety of cases are explored and any remaining problems are discovered. Thus, any bugs can be dealt with during the development phase, rather than discovering them when the system is being actively used by students.

Finally, over the course of implementing and running the two pilot sessions, we have come up with several technical recommendations. These recommendations relate to hardware, software, and other practical details of running a VW-based simulation.

1. *Test the computing environment in which the VW will be used* (e.g., a campus computer lab) before asking students to use the system. While a VW such as SL does not require high-performance computers, it *does* require special software, an up-to-date graphics card and a reasonably fast Internet connection. Furthermore, depending on the type of simulation being run, speakers, headphones and a microphone may also be required, which may not be available in a typical computer lab. Thus, this equipment may need to be provided by the simulation developer or facilitator, and if so, they will need to be tested with the computers in the lab to ensure compatibility.
2. *Provide all VW users – students, instructors and facilitators – with training in the virtual environment before conducting the simulation*. Most users will be unfamiliar with the VW interface and will thus require some training before they are comfortable using the VW system.
3. Whenever possible, *provide users with computers, accounts and avatars that are ready for use with the simulation*. Asking users to install software, create accounts and establish avatars will be an annoyance to all, but the most enthusiastic participants, and can be a significant barrier to participation for technologically inexperienced users.

### Discussion

The pilot sessions revealed two key themes. First, the VW provided learners with opportunities to communicate with other disciplines, which they would not have otherwise had until in clinical practice. Second, the technical issues experienced were distracting for the first group of participants when they tried to communicate information. However, despite these technical challenges, the participants felt the experience was extremely valuable. The pilot sessions demonstrated the successful application of a framework for the development of a student-focused IP module for paramedics and a trauma team in a VW.

The next steps for this project are to further develop the VW, particularly in the trauma room, and to work out some remaining technical glitches. We are also working on establishing a proper debriefing process for a virtual environment to ensure team communication and collaboration skills are discussed, rather than focusing on technical or novelty factors of the environment. We plan to expand the pilot to include a more comprehensive delivery of the module so that we can conduct proper educational research and determine the educational value of this work. Finally, we hope to include participants from a rural environment so that the physical distance is a real challenge and not easily overcome by a short car ride. The opportunities for rurally educated students – who traditionally had limited access to peer IP educational experiences – may be expanded with VWs. Further research is needed to determine the ability of these environments to enrich the learning experiences for students in rural environments.

A critical feature of introducing VWs into health science education is the evaluation of the student's experience. Research must determine the pedagogical value of VWs for the development of clinical and team skills, particularly among disciplines that do not typically work together before clinical practice. Future research to understand the pedagogical value of VWs may include investigation into two domains: social and environmental. The social domain can examine elements related to social presence, both individual and group, within the environment. For example, to what degree do students feel present within the environment? Body language or signals are limited within a VW to simple gestures and actions – this limited non-verbal communication capability may impact the student's perception of self within the VW and the behavioural interactions between avatars (24, 25). In addition, the personality of learners (26) and the student's learning style may influence the pedagogical value of the VW.

Within the environmental domain, an important consideration is the degree of fidelity required in replicating the real-world environment in a VW. For example, Dieckmann (27) reports that greater realism does not translate into improved achievement of learning objectives, which therefore raises the question of how ‘real’ something has to be for immersion to occur. Ecological validity, the degree to which the simulated experience relates to the actual clinical experience, is another important factor (27). While evidence suggests that a student's environment influences their learning (28), further investigation is required to understand the degree to which realism and ecological validity of a simulation influence a student's learning within an IP team.

Simulation-based training can be an effective means of delivering IP education (7). Strategies for effectively developing and delivering a simulation-based training program include using an established model for development, providing the necessary technological and administrative support and ensuring that the educators involved are committed and well-trained in the simulation technology (7). Indeed, in our experience, we have found that these elements can have a significant impact in the success of a VW simulation training program.

## Conclusion

This paper describes the implementation of a VW simulation for IP health science education. The application of VW technology for education and training in the health sciences is still in its infancy. The de Freitas and Oliver framework was shown to effectively support the design and development of VW simulations for IP education. The results from two pilot studies indicate that a VW simulation can be successfully implemented to support and enhance teaching and learning in health sciences. VW simulations are easy to reuse and to educate students for rare events or when they are geographically dispersed. As the capabilities of these technologies evolve, the potential for their use in health education will increase.
